# Pet acquisition trends and veterinary care access in the US

**DOI:** 10.1371/journal.pone.0325075

**Published:** 2025-07-02

**Authors:** Courtney Bir, Kayla Pasteur, Nicole Widmar, Candace Croney

**Affiliations:** 1 Department of Agricultural Economics, Oklahoma State University, Stillwater, Oklahoma, United States of America; 2 Department of Comparative Pathobiology, Purdue University College of Veterinary Medicine, West Lafayette, Indiana, United States of America; 3 Department of Agricultural Economics, Purdue University, West Lafayette, Indiana, United States of America; 4 Center for Animal Welfare Science, Departments of Comparative Pathobiology and Animal Science, Purdue University, West Lafayette, Indiana, United States of America; National Veterinary Research Institute (NVRI), NIGERIA

## Abstract

The COVID-19 pandemic presented a host of unique challenges for individuals worldwide, particularly for pet owners, due to widespread shutdowns, social distancing, and financial stress. While pet acquisition increased during this time, the impact on veterinary care access and pet ownership trends remain underexplored. Within the online survey of 751 US residents 79% were pet owners (n = 596). Twenty percent of all pet owners reported difficulty accessing basic veterinary care, such as vaccinations or annual exams. Logit models revealed that having children and working from home increased the likelihood of acquiring a pet during the pandemic. Additionally, owning a pet acquired during the pandemic and managing pets with behavioral issues were associated with greater challenges in accessing veterinary care. These findings highlight unique circumstances during COVID-19 related to pet acquisition and veterinary care, which may be expanded to other situations. A better understanding of these difficulties is essential to develop solutions that protect animal welfare and support the human-animal bond, particularly in times of crisis.

## Introduction

The bonds that are formed between people and animals have been demonstrated to provide benefits to both, with noteworthy physical, social, and emotional benefits accruing for humans [1,2], despite some inconsistent findings [3]. Not surprisingly, the prevalence of pet ownership in the United States is high. According to the American Pet Products Association, 58 million US households keep one or more dogs and 40 million keep at least one cat [4]. For those who keep pets, the majority often characterize them as family members [5–7] and projections for spending on their food and treats are estimated at $66.9 billion [4].

Given that pets provide a source of enjoyment in the household [8] as well as stress buffering and social support [9,10], it makes sense that many people relied on their existing pets or sought to acquire one during the COVID-19 pandemic to help them cope with global shutdowns, social distancing, and overall panic [11]. According to Bowen et al. [12], the quality of life experienced by pet owners during the pandemic was strongly influenced by the emotional effects of confinement. However, pets played a major role in mitigating the effects of negative emotional states.

A great deal of research has been conducted on the sources people choose when acquiring a pet, and the reason for their choice of pets, including characteristics of interest, and source of acquisition [5,13,14]. For most of the past decade, the percentage of pets acquired purposely (i.e., adopted or purchased) has been increasing, while the proportion of pets acquired incidentally (i.e., gifted or found as a stray) has been decreasing [15]. Some aspects of this paradigm shifted with the COVID-19 pandemic, along with the changing health and social guidelines. Initially, public interest in adopting a new pet spiked in many regions of the world [16,17], far above the preceding 5-year average. However, not every region or household saw this type of increase [18]. For example, some people may have opted against pet adoption at this time because of concerns about their pets contracting or transmitting COVID-19 to them [19], while others could not afford to bring a new pet into the home. In addition to the general increase in pet acquisition, the number of dogs and cats purchased from a breeder or pet store increased while the number adopted from a shelter or rescue decreased [20]. This might have been due in part to the decreased number of animals available in shelters during the early phases of the pandemic [21] as increased public demand for pets may have driven increases in adoption and reduced availability of pets through shelters and rescues. By December 2020, interest in dog adoption had dropped back to the level of the previous 5-year average, while interest in cat adoption has remained high since [17].

Despite the number of studies documenting the emotional and psychological benefits of pet ownership during the pandemic, not all outcomes were positive. Factors such as access to resources needed to support the needs of pets heavily contribute to whether or not pet ownership is successful in benefiting the owner under the circumstances [22,23]. In addition, access to veterinary care potentially constrains people’s ability to provide appropriate care for and ultimately, to keep their companion animals. Prior to the COVID-19 pandemic, the need to address gaps in access to veterinary care had been identified, as this potentially compromises animal health and overall welfare. Inability to readily access veterinary care directly impacts pet owners from underserved, disabled, and low-income communities [24–26]. The onset of a global pandemic further exacerbated the issue of affordability and accessibility of veterinary care, particularly for pet owners with disabilities [22,27–30].

Understanding the impacts of the COVID-19 pandemic on pet ownership, acquisition, care, and retention may provide key insights about behaviors and decision-making processes of pet owners and the different factors that influence these. Such knowledge may be beneficial in helping to identify and envision interventions for education and support for pet owners that better support their health and well-being as well as the welfare outcomes of the animals they keep. Therefore, this study sought to [1] identify who had and/or acquired a pet during the COVID-19 pandemic, [2] understand the experiences of pet owners during the COVID-19 pandemic, particularly their experiences with accessing veterinary care, and [3] determine whether there was variation in pet owners’ experiences during the COVID-19 pandemic related to demographics. We hypothesized that the novel experiences of pet owners under the unique circumstances of a global pandemic (i.e., social distancing, isolation) would differ depending on the availability and accessibility of different resources such as time, finances, and access to veterinary care.

## Methods

### Survey design and summary statistics

The online survey tool, Qualtrics [31], was used to gather information from 751 U.S. residents who were recruited to participate in the study from November 30, 2021 to December 8, 2021. Purdue and Oklahoma State University researchers developed, pre-tested, and designed the survey to collect the following data: [1] demographics, [2] work from home preferences, and [3] changes in personal behavior related to working from home. The sample was targeted to be representative of the U.S. population in terms of sex, age, income, education, and geographical region of residence [32–35]. Regions of residence were defined as in the Census Bureau Regions and Divisions [36]. Kantar [37], who hosts a large opt-in panel of potential participants, was used to obtain survey respondents. To be included in this study, respondents were required to provide written consent by selecting “I agree to participate in this study” and indicate that they were 18 years of age or older. Although the objectives of this research were related to pet acquisition, official demographic information regarding those who own pets are not available. By targeting the US population, we should capture appropriate demographic representation of pet ownership as well. Furthermore, one objective was to determine pet acquisition during the COVID-19 pandemic, given the possibility that a pet was acquired by a first-time pet owner, and then later relinquished, making them a non-current pet owner, it was important to target a population beyond current pet owners.

The entire survey comprised approximately 30 questions with randomized response options and was designed to be completed in 25 minutes or less. To ensure brevity and minimize survey fatigue, Qualtrics’ display logic was utilized to tailor future questions based on respondents’ previous answers. For example, all respondents (n = 751) were asked about personal behavioral changes pre and post March 2019, when many areas instituted COVID-19 lockdowns. However, only respondents who worked (n = 435) were asked specific questions regarding work location preferences. Additionally, participants who indicated that they did not own pets were not asked to respond to subsequent questions related to pet ownership during the COVID-19 pandemic. All questions relevant to this study are provided in supplementary material. Summary tables and percentage frequencies were calculated for all categorical variables. To determine how close the sample was to the US census, chi-square test of proportions was conducted to compare the proportions of demographic variables in the sample to that of U.S. Census data.

### Logit models

Two logit models were employed to evaluate who obtained a pet during the COVID-19 time period, and if pet owners had difficulty obtaining veterinary care. Logit models were chosen because the probability the respondent obtained a pet during the COVID-19 time period and whether they experienced difficulties accessing basic or specialty veterinary care took on the form of 1 or 0. The model for obtaining a pet during the COVID-19 time period can be represented by the equation:


$${ObtainPet}_{n}=\beta_{1}{Male}_{n}+\beta_{2}{Age}_{n}+\beta_{3}{Income}_{n}+\beta_{4}{Region}_{n}+\beta_{5}{Child}_{n}+\beta_{6}{WorkHome}_{n}+\varepsilon .$$ (1)

Where *Male*_*n*_ indicates the sex of respondent *n* and is in reference to female, *Age*_*n*_ is each of the age categories as defined by the U.S. census and are in reference to the category 66 and over, *Income*_*n*_ is the household income categories as defined by the U.S. census and are in reference to the income category $100,000 or greater, *Region*_*n*_ is the region of residence which are in reference to the region South. *Child*_*n*_ indicates the household has a child, *WorkHome*_*n*_ indicates the respondent sometimes or always worked from home during the period of November 30, 2021 to March 2022, and ε is the error term.

To account for first-time pet owners who acquired their pet during the COVID-19 pandemic and may have relinquished their pets, all respondents were asked if they obtained a pet during this time period, and the full sample—including current and former pet owners—was used in the regression. Although official consistent publicly available demographics information is not available regarding pet ownership, there are general trends. For example, Applebaum et al. found that women and higher income households were more likely to have a dog [38]. Additionally, younger people were more likely to have any pet [38]. It was unknown if the procurement of pets during COVID-19 would reflect the same as general pet ownership so demographic variables were included. Households with children are more likely to have pets [39]; however, given the additional burdens faced by families with children during the COVID-19 pandemic, it was unclear if having children in the household would negatively or positively impact pet procurement [40]. Similarly, it was unclear whether working from home would result in the desire to obtain a pet.

Veterinary difficulties were defined as having difficulty accessing basic or specialty veterinary care. The model for veterinary difficulties can be represented by the equation:


$$\mathrm{\backslash [}{VetDifficulty}_{n}=\beta_{1}{Male}_{n}+\beta_{2}{Age}_{n}+\beta_{3}{Income}_{n}+\beta_{4}{Rurality}_{n}+\beta_{5}{Dog}_{n}+\beta_{6}{Cat}_{n}+\beta_{7}{COVIDPet}_{n}+\beta_{8}{Training}_{n}+\beta_{9}{Anxiety}_{n}+\beta_{10}{Child}_{n}+\beta_{11}{WorkHome}_{n}+\varepsilon .\mathrm{\backslash ]}$$ (2)

Where *Male*_*n*_, *Age*_*n*_, *Income*_*n*_, *Child*_*n*_, *WorkHome*_*n*_ are as defined above. *Rurality*_*n*_ is a continuous variable as defined by the USDA from a scale of 1 (urban) to 9 (rural). The variable *Dog*_*n*_ indicates the respondent has a dog, the variable *Cat*_*n*_ indicates the respondent has a cat, and *COVIDPet*_*n*_ indicates the respondent got a pet during the COVID-19 time period. The variable *Training*_*n*_ indicates the respondent experienced increased need for training due to COVID-19 isolation, and *Anxiety*_*n*_ indicates the respondent’s pet experienced increased separation anxiety when left at home, and ε is the error term the error term.

Use of veterinary care services differs based on demographic factors. Neill et al. found that males and older pet owners spent less time at the veterinary clinic [41], while Bir et al. found that age and income increased the probability of visiting the veterinarian [42]. It was likely that demographic factors would also influence difficulty accessing veterinary care, so they were included in the model. Being a cat owner decreases the likelihood of seeking veterinary care [43], so variables for cat and dog ownership were included. Shortages of rural veterinarians have been well documented, and therefore rurality was included [44]. Acquiring a pet during the COVID-19 pandemic could have coincided with issues finding veterinarians willing to takenew patients. Therefore, the variable 'training' served as a proxy forneed for support with both training of pets and pet behavior, including behavioral issues. These needs can influence stress surrounding veterinary visits and may require more specialized care, especially for pets that are anxious in homes or at the veterinary clinic. Respondents with children may have faced additional barriers related to having children at home during the pandemic. It was unclear the impact working from home would have on veterinary access, as it may result in more or less flexibility in scheduling given the individual circumstance.

## Results

A total of 1888 individuals opened the survey link provided to them by Kantar. Of those, 751 completed the survey resulting in 39.7% completion rate. Table 1 presents demographics for the 751 respondents to the survey. All demographics were proportionally comparable to the U.S. population, according to U.S. Census data [32–35], except for the proportion of participants aged 25–34, those with incomes of $100,000 or higher, those who did not graduate from high school, and those who earned an associate or bachelor’s degree.

**Table 1:** Demographic characteristics of respondents (n = 751).

Demographic Variable	Percentage of Respondents	Pet Owners^1^	US Census
	N = 751	N = 596	
*Gender*			
Male	46	32a^2^	49
Female	54	38b	51
*Age*			
18-24	13	9a	12
25-34	14ψ	12ab	18
35-44	17	14b	16
45-54	17	12ab	16
55-65	18	13bc	17
65 +	22	10ac	21
*Income*			
$0-$24,999	20	12a	18
$25,000-$49,999	23	16b	20
$50,000-$74,999	19	14ab	17
$75,000-$99,999	14	10a	13
$100,000 and higher	23ψ	18b	31
*Education*			
Did not graduate from high school	4ψ	3a	11
Graduated from high school, Did not attend college	28	19b	27
Attended college, No degree earned	22	14c	21
Attended college, Associates or bachelor’s degree earned	32ψ	24d	29
Attended college, Graduate or professional degree earned	14	10e	13
*Region of residence*			
Northeast	18	13a	17
South	37	27b	38
Midwest	22	14a	21
West	22	15a	24

ψ Indicates the percentage of respondents is statistically different than the U.S. census at the 0.05 level.

^1^ Pet owners are defined as those who currently have at least one pet, or had a pet in the last two years but not currently

^2^ Matching letters indicate the percentage of respondents within that demographic category are not statistically different. Non-matching numbers indicates the percentage of respondents are statistically different. For example, the percentage of females with pets is statistically different than the percentage of men. The percentage of those 18–24 with pets is not statistically different than the percentage aged 25–34.

Of those who responded (n = 751), 79% were pet owners (n = 596). A pet owner was defined as those who currently have at least one pet, or had a pet in the last two years but not currently. The test of proportions was conducted to determine statistical differences amongst the demographic variables within pet owners. A higher percentage of women were pet owners when compared to men. There were no clear differences amongst age groups. A higher percentage of respondents with an income of $100,000 and higher when compared to those with an income of $0-$24,999 and those with an income of between $75,000-$99,999 had pets. A lower percentage of respondents who did not graduate from high school were pet owners when compared to all other education levels. A higher percentage of respondents from the south were pet owners when compared to all other regions.

**Fig 1:**
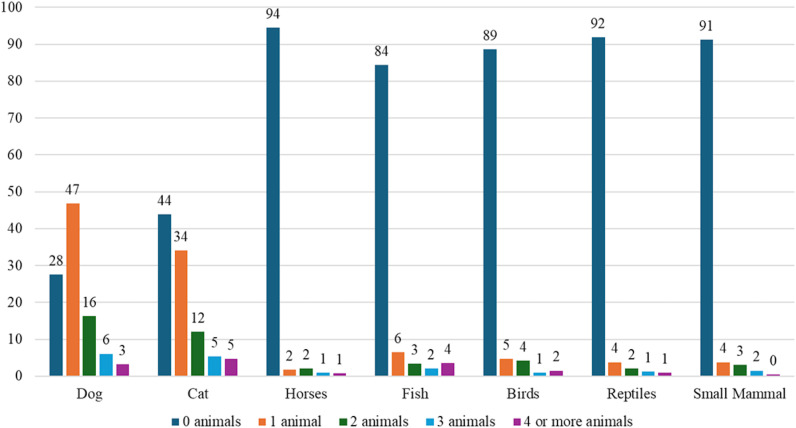
**Number of each type of pet owned by respondent, percentage of pet owners (n ** = **526).**

Considering the full sample (n = 751) 51% of respondents owned at least one dog, 39% owned at least one cat, 4% owned at least one horse, 11% owned fish, 8% owned birds, 6% owned reptiles, and 6% owned small mammals. Fig 1 illustrates the type and number of pets each pet owning respondent kept.

Table 2 presents the experiences of pet owners related to pet keeping and care during the COVID-19 pandemic. Cat and dog owners both reported an increased need for training of their pets due to social isolation or inability to socialize their pets during the pandemic. However, the percentage of those reporting these needs was higher (35%) for those who obtained a pet during the pandemic, when compared to those who acquired their pet before COVID-19. This difference was even more pronounced for the statement, “my pet experienced increased anxiety when left at home”, with only 16% of pet owners who had acquired a pet before COVID-19 indicating yes, compared to 42% of pet owners who acquired a pet during COVID-19. Twenty percent of pet owners in general indicated they had difficulty accessing basic veterinary care, including vaccines and/or annual exams. However, 34% of pet owners who acquired pets during COVID-19 experienced this difficulty while only 14% of pet owners who had acquired their pet before the pandemic experienced it. Similar findings occurred for difficulty accessing specialty veterinary care and experiencing extended waits for veterinary care appointments. For instance, though 29% of pet owners overall reported they experienced extended waits for veterinary care appointments, a much higher proportion (42%) of pet owners who acquired their pet during COVID-19 experienced these extended waits than those who had obtained their pets before the pandemic began.

**Table 2:** Pet owner experiences during the COVID-19 pandemic time period. N given in table, percentage of respondents.

	Pet owners	Dog Owners	Cat Owners	Pet Acquired During COVID	Pet Acquired Before COVID
	N = 526	N = 204	N = 118	N = 175	N = 351
Increased need for training due to isolation or lack of socialization	19	13	8	35	11
Pet experienced increased separation anxiety when left at home	24	24	19	42	16
Difficulty accessing basic veterinary care including vaccinations and/or annual exams	20	17	8	34	14
Difficulty accessing specialty veterinary care	19	15	6	33	11
Extended waits for veterinary care appointments	29	27	15	42	22

**Fig 2:**
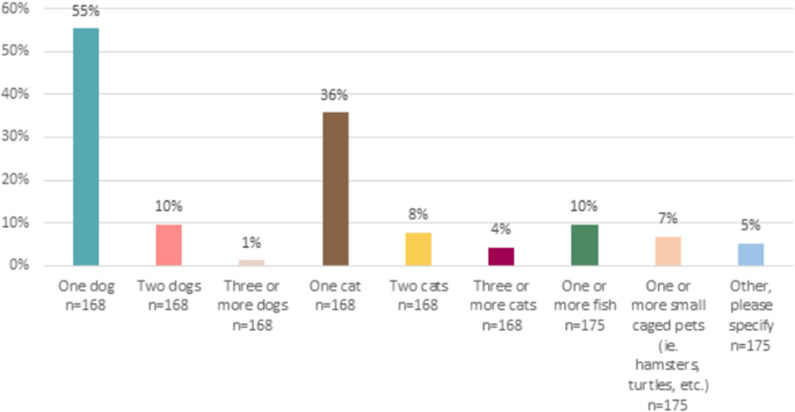
Number and type of pet obtained by respondents during COVID-19. Percentage of respondents who obtained a pet during COVID-19.

Of those who acquired a pet during the pandemic, the majority (55%) brought home a dog. The next highest percentage (36%) acquired a cat (see Fig 2). Except for dogs and fish, fewer than 10% of respondents obtained more than one pet of each type listed. Note that within Fig 2, seven dog owners and seven cat owners selected multiple options and were therefore not included.

Table 3 presents ways in which respondents acquired a companion animal during the COVID-19 pandemic, accessibility of the pets of people’s choice, the status of the pets acquired and reasons for relinquishing newly acquired pets. The majority adopted (41%) from a shelter or rescue. The vast majority (73%) were able to obtain the pets of their choosing; however, a quarter of respondents had already rehomed their newly acquired pet by December 2021. Behavioral problems (55% overall) represented the highest proportion of the reasons given for relinquishing a pet obtained during the pandemic followed by health issues (45% overall), and costs of maintaining the pet (25%).

**Table 3:** COVID-19 pet acquisition, accessibility, and status of pets acquired. Percentage of respondents, n indicated in table. Note multiple selections were allowed.

	All pet types
	N = 175
*Way Respondent Acquired new pet*	
Adoption (shelter or rescue organization)	41
Bred them myself	9
Purchased from a breeder	18
Purchased from pet store	26
Stray	15
Gift from family member/friend	15
Other	7
*Ability to obtain the pet they wanted during the COVID-19 era*	
Yes, I was able to access the pet I wanted	73
I did not have a specific pet in mind	22
I would have made a different choice if there was greater availability/access	5
Other	5
*Status of pets acquired*	
I have already given away or otherwise rehomed my new pet	25
My new pet has passed away	12
Yes, I plan on keeping my new pet	55
No, I plan on rehoming or giving away my new pet in the future	4
Other	3
	**All pet types** ^**1**^ **N = 51**
*Reasons for relinquishing pet*	
Behavioral difficulties	55
Health difficulties	45
Too costly	25
Cannot manage necessary pet care	14
Other	6

^1^Sample includes only those who indicated they had already or planned on relinquishing their pet

In the regression model exploring factors related to obtaining a pet during the COVID-19 period, being aged 18–24, 25–34, and 35–44 all increased the likelihood of obtaining a pet during COVID when compared to those 66 and older (Table 4). Additionally, having an income of $0-$24,999 decreased the likelihood of obtaining a pet during the pandemic when compared to those with an income of $100,000 or greater. Being from the Northeast and Midwest decreased the likelihood of obtaining a pet during the COVID-19 period when compared to the South. Having a child in the household and working from home both increased the likelihood of obtaining a pet during COVID-19.

**Table 4:** Logit model of pet acquistion during the COVID-19 time period (N = 751). Model is statistically significant at <0.000.

Variable	Odds Ratio	SE	Marginal
Male^1^	0.850	0.168	−0.025
*Age* ^2^			
18-24	2.941**	1.195	0.165 **
25-34	2.712**	1.106	0.152 **
35-44	2.669**	1.067	0.150 **
45-54	1.314	0.529	−0.042
55-65	1.023	0.426	−0.004
*Income* ^3^			
$0-$24,999	0.506**	0.165	−0.104**
$25,000-$49,999	0.879	0.249	−0.020
$50,000-$74,999	0.724	0.2114	−0.049
$75,000-$99,999	0.695	0.206	−0.055
*Region of residence* ^4^			
Northeast	0.615*	0.168	−0.074*
Midwest	0.644*	0.171	−0.067*
West	0.827	0.205	−0.029
Has a child in the household^5^	2.336***	0.533	0.130***
Work/worked from home^6^	1.685*	0.378	0.080**
Constant	0.148	0.056	

^1^ Male variable is in reference to the variable female.

^2^ Age variables are in reference to the dropped category 66+.

^3^ Income variables are in reference to the dropped category $0–24,999.

^4^ Region variables are in reference to the dropped category South.

^5^ Has a child is in reference to not having a child in the household.

^6^ Defined as sometimes or always worked from home March 2020 to November 30, 2021; only respondents who worked in the past 2 years (n = 435).

* Statistically significant at the 0.10 level, ** at the 0.05 level, *** at the 0.001 level.

Difficulty obtaining basic or specialty veterinary care services during the COVID-19 period was significantly associated with being between the ages 25−44, acquiring a pet during the COVID-19 pandemic, having a dog, having a pet needing training, and a pet experiencing separation anxiety when left alone at home (Table 5). Being male, income level, rurality, having a cat, having a child, and working from home did not have a statistically significant effect.

**Table 5:** Logit model of veterinary difficulties for all pet owners (n = 526). Veterinary difficulties were defined as having difficulty accessing basic or specialty veterinary care.

Variable	Odds Ratio	SE	Marginal
Male^1^	1.488	0.374	0.055
*Age* ^2^			
18-24	1.977	1.163	0.094
25-34	3.090*	1.788	0.155*
35-44	2.492	1.437	0.126
45-54	2.206	1.257	0.109
55-65	1.873	1.058	0.086
*Income* ^3^			
$0-$24,999	0.605	0.259	−0.069
$25,000-$49,999	0.730	0.267	−0.043
$50,000-$74,999	1.006	0.350	0.001
$75,000-$99,999	0.974	0.366	−0.004
Rurality^4^	1.027	0.053	0.004
Has a dog	2.236**	0.718	0.111**
Has a cat	1.092	0.276	0.012
Got a COVID pet	1.973**	0.492	0.094**
Experienced increased need for training due to COVID-19 isolation	5.240***	1.523	0.228***
Experienced increased separation anxiety when leaving pets at home	2.816***	0.767	0.143***
Has a child in the household^5^	0.772	0.231	−0.036
Work/worked from home^6^	1.425	0.409	0.049
Constant	0.026***	0.016	

^1^ Male variable is in reference to the variable female.

^2^ Age variables are in reference to the dropped category 66+.

^3^ Income variables are in reference to the dropped category $0–24,999.

^4^ Rurality as defined by the USDA as 1 (urban) to 9 (rural).

^5^ Has a child is in reference to not having a child in the household.

^6^ Defined as sometimes or always worked from home March 2020 to November 30, 2021; only respondents who worked in the past 2 years (n = 435).

* Statistically significant at the 0.10 level, ** at the 0.05 level, *** at the 0.001 level.

## Discussion

This study examined the novel experiences of U.S. pet owners and the unique challenges they faced during the global COVID-19 pandemic. Our hypothesis was generally met. The findings offer valuable insights into how pet owners’ demographic characteristics influence their acquisition of pets and their experiences as pet owners. This information is important as it may shed light on factors that may subsequently inform pet relinquishment decisions, particularly in situations when difficulties in accessing veterinary care arise.

Unsurprisingly, many households obtained pets during the early stages of the pandemic. While reasons for acquiring a pet at this time may have varied, the perceived benefits of pet ownership to people’s social, emotional, and mental health likely factored into people’s decision-making. As one study reported [45], UK pet owners who felt they benefited from the presence of their pet reported poorer mental health before the pandemic began. Recent studies have suggested that during social distancing, pets may have buffered some of the stressors of working from home without normal social interactions with other people [20]. Conversely, Hoffman et al.[20], found that dog and cat ownership did not increase during the pandemic, partly because it was an economically unstable period similar to the Great Recession of 2008.

Our findings indicate that respondents, especially those who acquired pets during COVID had an increased need for training due to isolation or lack of socialization, and their pets experienced separation anxiety when left at home. Others found that pet owners experienced training issues and difficulty finding basic supplies for their pets [28]. More respondents with a dog expressed this need. Dogs are often perceived to have greater training needs in comparison to other companion animals. The inability to meet a pet’s needs during or outside of a pandemic may create distress for pet owners and their families. In fact, having the responsibility of pet ownership while experiencing significant hardship has been reported to be detrimental to the mental and emotional wellbeing of the owner [27,28,46].

The majority of people looking for pets during COVID were able to obtain the pet of their choosing. Cost has previously been shown to be a factor in new owners’ decisions about the way they acquire a pet [47]. In fact, economic recessions have historically resulted in a decrease in dog and cat adoptions due to concerns about ability to afford supplies, veterinary care, and other costs associated with pet ownership [48]. The majority of respondents chose to adopt from a shelter or rescue, which is often a lower cost option, but our results cannot shed further light on whether this choice was a preference, or due to other factors such as cost, or availability.

A quarter of pets obtained during COVID were rehomed. Hoffman et al. reported a smaller percentage (12%) of owners relinquished a pet during the COVID-19 pandemic [20], this is still double the 6% who reported rehoming their pet(s) before the pandemic began [49]. The difference in percentage may be due to our further specification that the pet also had to be obtained during COVID. The most selected reason for relinquishment was behavior difficulties, followed by health difficulties. Pet ownership during the pandemic presented considerable challenges for some people [28]. For example, some studies found that people with pets and/or children reported higher levels of distraction while working from home [50]. Twenty-five percent of respondents relinquished pets due to cost. As with pet acquisition, financial concerns are often a key determinant in decisions to relinquish pets, with some households likely experiencing exacerbated challenges due to financial constraints associated with the pandemic [20,48]. Carroll et al. found financial constraints were the most frequently reported reason for both giving up a pet and considering giving up a pet during COVID [51]. Pet relinquishment is an important issue to understand because pets surrendered to a shelter may be more likely to be euthanized than rehomed with a new owner [52].

In the logit model of pet acquisition during COVID, being in the lowest income bracket ($0-$24,999) decreased the likelihood of obtaining a pet. Cost has previously been shown to be a factor in new owners’ decisions about the way they acquire a pet [47]. In fact, economic recessions have historically resulted in a decrease in dog and cat adoptions due to concerns about ability to afford supplies, veterinary care, and other costs associated with pet ownership [48].

Having a child in the household and work/worked from home also increased the likelihood of pet acquisition. It is plausible that working from home, especially if there were children in the home, might have encouraged people to believe they would have more time to give a pet. Some may have believed it would be beneficial to their children to have a pet during this time either to help occupy them or to help with pet care such as feeding, exercise, and play. Others may have decided it was ideal to introduce a new pet during a time when all family members were homebound together and could theoretically give the pet even more time and attention than they might otherwise have received.

Interestingly, in the logit model of veterinary access difficulties, income was insignificant. Even before the COVID-19 pandemic, a commonly cited challenge for pet owners seeking appropriate medical care was cost [53]. Financial hardships may have been exacerbated during the pandemic when there was both a recession, and for some workers, greater income insecurity, resulting in newly vulnerable populations [25]. Previous studies [42], found that those with higher income were more likely to go to the veterinarian. Our finding, may also be a reflection of this. Those with lower incomes may not have experienced difficulty accessing care because they did not seek it.

Having a pet acquired during the COVID-19 pandemic was associated with increased difficulty of accessing veterinary care. Inability to access veterinary care, especially for newly acquired pets, as occurred during the pandemic, is highly problematic given the critical role veterinarians play not just in supporting pet health but also the human-pet bond. It is possible that due to health concerns, related labor shortages, and consequent demands for their time and expertise, some veterinarians may have been unwilling or unable to take on new patients. Many veterinary hospitals and clinics, adapting to an airborne illness, took steps to prevent the spread of COVID-19 among their workers and prioritized urgent care for companion animals. This typically had the effect of reducing efficiency and creating a backlog for clients seeking basic wellness care for their pets [54]. Given expectations that veterinarians are the go-to resources for animal welfare support beyond just health, the ability to access a veterinarian prior to acquiring a pet offers an important safeguard against making choices that might result in a mismatch between people’s expectations of the pet they are considering, their ability to maintain the pet, their choice in where to source a pet, and their knowledge about all of the additional services and resources needed to support the pet in the home. Ensuring access to pet care in general, and particularly during times when the demand for pets peaks (as occurred during the COVID-19 pandemic) is essential for ensuring animal welfare.

There is a need for innovation in addressing challenges related to access to veterinary care. For example, although there is increasing awareness of changes to the industry that could increase access to veterinary care (e.g., telemedicine; [25]), few widescale changes have been implemented. This may have opened more opportunities for those with pets who had behavioral issues which increased difficulties accessing care in the model. Few laws, for instance, have been changed that would allow veterinarians to establish new patient relationships without in-person examination of an animal [55]. Given behavioral issues, alongside decreased capacity within the clinic, these issues are compounded. Other sources have predicted ongoing shortages of veterinarians available to provide care for adopted pets that may last for several years to come [56], suggesting that the difficulties pet owners face in obtaining veterinary care may continue.

## Conclusions

These findings highlight unique circumstances during the COVID-19 pandemic related to pet acquisition and veterinary care, which may be expanded to other situations. Although some pet owners had increased need for training due to isolation or lack of socialization, those who obtained a pet during the pandemic had a greater need. These pets also experienced greater separation anxiety, and their owners had additional issues accessing veterinary care. This was further demonstrated by the 55% or respondents who relinquished their COVID-acquired pet doing so for behavioral issues. Working from home and having a child in the household both increased the likelihood of acquiring a pet during the COVID-19 pandemic, but had no impact on pet owners' difficulty in accessing veterinary care. Experiencing increased need for training and other pet behavior support, in particular addressing behavior problems such as separation anxiety that require specialized care, were related to difficulty in accessing veterinary care. Although it is likely the COVID-19 pandemic magnified motivations and stressors associated with obtaining pets and meeting their needs, this information is helpful in underscoring the need for the veterinary community to develop support systems for pet owners. For example, veterinarians may consider investing time and resources into providing more routine behavioral support for clients, or they might adopt more low stress handling techniques within their clinics.

Although we strove to be nationally representative, the sample was statistically different for one age category, one income category, and two education categories. Ideally, we would be able to compare the representativeness of pet ownership, but due to a lack of standardized sampling, and proprietary data, a good measure of pet ownership is not available [38]. Although we began to explore the complexities of pet ownership and veterinary access, future researchers can build from this work. A deeper qualitative analysis of the human pet interaction would be a possible extension of this paper.

Given these findings, more research needs to be conducted to examine why pet ownership may have negative impacts under some circumstances and how we can address those issues. Additionally, we must further explore the factors related to difficulty in accessing veterinary care (i.e., age, sex, type of pet) to develop targeted interventions that will better support vulnerable pet owners. These efforts will be crucial in minimizing pet relinquishment, preserving the human-animal bond, and safeguarding animal welfare. Last, there is a need to examine alternatives to enhancing and increasing access to veterinary care, in general and under emergency conditions. Our findings reiterate that the veterinarian’s role in providing healthcare and other forms of essential support including owner/pet family education are essential to informing public decision-making that can help ensure animal welfare and the human-animal bond.

## Supporting information

S1 File Survey Questions. (DOCX)

S2 File Survey Dataset. (XLSX)
