# Supplementary material for: Pet acquisition trends and veterinary care access in the US
Source: PLoS One. 2025 Jul 2;20(7):e0325075. doi: 10.1371/journal.pone.0325075 (PMC12220994; doi:10.1371/journal.pone.0325075)
Supplement: S1 File — (DOCX) [file pone.0325075.s001.docx]

Questions Analyzed

**Participants were presented with the research participant consent form approved by IRB.**

Q1.4 I am:

- Male (1)
- Female (2)

Q1.5 I am _____ years old.

- Under 18 (1)
- 18 - 24 (2)
- 25 - 34 (3)
- 35 - 44 (4)
- 45 - 54 (5)
- 55 - 64 (6)
- 65 + (7)

Q1.6 My household (including myself, all other adults and any children) has the following number of members. (Please enter a "0" for children if your household does not contain any members under 18 years of age.)

- Adults (18 years and older) (1) ________________________________________________
- Children, Aged 12 - 18 years (2) ________________________________________________
- Children, Aged 5 to 11 years (3) ________________________________________________
- Children, Under 5 years of age (4) ________________________________________________

Q1.7 My annual pre-tax household income is:

- $0-$24,999 (1)
- $25,000-$49,999 (2)
- $50,000-$74,999 (3)
- $75,000-$99,999 (4)
- $100,000 and higher (5)

Q1.8 The best description of my educational background is:

- Did not graduate from high school (1)
- Graduated from high school, Did not attend college (2)
- Attended College, No Degree earned (3)
- Attended College, Associate's or Bachelor's Degree earned (4)
- Attended College, Graduate or Professional Degree earned (5)

Q1.9 My region of residence is: ___________. Select one option from the drop down menu.

- Northeast (CT, ME, MA, NH, NJ, NY, PA, RI, VT) (1)
- South (AL, AR, DE, DC, FL, GA, KY, LA, MD, MS, NC, OK, SC, TN, TX, VA, WV) (2)
- Midwest (IL, IN, IA, KS, MI, MN, MO, NE, ND, OH, SD, WI) (3)
- West (AK, AZ, CA, CO, HI, ID, MT, NV, NM, OR, UT, WA, WY) (4)

Q1.14 Do you or anyone in your household have a pet now, or have you had a pet in the last 2 years?

- Yes, my household has at least one pet currently (1)
- Yes, my household has had a pet in the past 2 years but not currently (2)
- No, my household has not had a pet at any point in the last 2 years (3)

Q1.15 Are you currently employed, had any form of employment or worked for pay during the past 2 years?  


Please answer yes if you are full-time, part-time, or have been occasionally employed at any point in the past 2 years.

- Yes (1)
- No (2)

Q2.2 Please indicate your work locations for the time periods outlined:

|  | I worked from home | | | I worked in an office or other place of employment | | | I have a physical place of employment (i.e. office) but am able to work remotely | | |
| --- | --- | --- | --- | --- | --- | --- | --- | --- | --- |
|  | Never (1) | Sometimes (2) | Always (3) | Never (1) | Sometimes (2) | Always (3) | Never (1) | Sometimes (2) | Always (3) |
| Before March 2020 (1) |  |  |  |  |  |  |  |  |  |
| March 2020 - May 2020 (2) |  |  |  |  |  |  |  |  |  |
| June 2020 - August 2020 (4) |  |  |  |  |  |  |  |  |  |
| September 2020 - December 2020 (5) |  |  |  |  |  |  |  |  |  |
| January 2021 - May 2021 (6) |  |  |  |  |  |  |  |  |  |
| June 2021 - August 2021 (7) |  |  |  |  |  |  |  |  |  |
| September 2021 - Today (8) |  |  |  |  |  |  |  |  |  |

Q4.1 Please select the number of each animal you have had in your household in the past 2 years:

|  | 0 (1) | 1 (2) | 2 (3) | 3 (4) | 4 or more (5) |
| --- | --- | --- | --- | --- | --- |
| Dogs (1) |  |  |  |  |  |
| Cats (2) |  |  |  |  |  |
| Horses (3) |  |  |  |  |  |
| Fish (4) |  |  |  |  |  |
| Birds (5) |  |  |  |  |  |
| Reptiles (6) |  |  |  |  |  |
| Small mammals (Mice, Guinea Pigs, Rabbit, Hamster, Rat, Chinchilla, Ferrets, Gerbil, etc.) (7) |  |  |  |  |  |

Q4.2 Have you experienced any of the following during the COVID-19 pandemic time period regarding your pets?

|  | Yes (1) | No (2) | Does Not Apply (3) |
| --- | --- | --- | --- |
| Increased needs for training due to isolation or lack of socialization (1) |  |  |  |
| Increased separation anxiety when leaving pets at home (2) |  |  |  |
| Difficulty accessing basic veterinary care, including vaccinations and/or annual exams (3) |  |  |  |
| Difficulty accessing specialty veterinary care (4) |  |  |  |
| Extended waits for veterinary care appointments (5) |  |  |  |

Q4.3 Have you obtained a new pet during the COVID-19 pandemic time period (between March 2020 and present day)?

- Yes (1)
- No (2)

Skip To: End of Block If Have you obtained a new pet during the COVID-19 pandemic time period (between March 2020 and pres... = No

Q4.4 What type of pet(s) did you obtain? (Check all that apply)

- One dog (1)
- Two dogs (2)
- Three or more dogs (3)
- One cat (4)
- Two cats (5)
- Three or more cats (6)
- One or more fish (7)
- One or more small caged pets (ie. hamsters, turtles, etc.) (8)
- Other, please specify (9)

Q4.5 How did you acquire your pet? (Check all that apply)

- Adoption (shelter or rescue organization) (1)
- Bred them myself (2)
- Purchased from a breeder (3)
- Purchased from pet store (4)
- Stray (5)
- Gift from family member/friend (6)
- Other, please specify (7) _____

Q4.7 Were you able to find and obtain the pet you wanted during the Covid-era? (Select all that apply)

- Yes, I was able to access the pet I wanted (1)
- I did not have a specific pet in mind (2)
- I would have made a different choice if there was greater availability/access (3)
- Other (4)

Q4.8 Have you kept and/or are you planning on keeping your new pet?

- I have already given away or otherwise rehomed my new pet (1)
- My new pet has passed away (2)
- Yes, I plan on keeping my new pet (3)
- No, I plan on rehoming or giving away my new pet in the future (4)
- Other (5)

Display This Question:

If Have you kept and/or are you planning on keeping your new pet? = I have already given away or otherwise rehomed my new pet

Or Have you kept and/or are you planning on keeping your new pet? = No, I plan on rehoming or giving away my new pet in the future

Q4.9 Why have you relinquished your pet or are you considering relinquishing your pet?

- Behavioral difficulties (1)
- Health difficulties (2)
- Too costly (3)
- Cannot manage necessary pet care (4)
- Other (5)
